# The adipocyte apolipoprotein M is negatively associated with inflammation

**DOI:** 10.1016/j.jlr.2024.100648

**Published:** 2024-09-19

**Authors:** Laurie Frances, Mikael Croyal, Soline Pittet, Léa Da Costa Fernandes, Milan Boulaire, Laurent Monbrun, Ellen E. Blaak, Christina Christoffersen, Cédric Moro, Geneviève Tavernier, Nathalie Viguerie

**Affiliations:** 1 Institute of Metabolic and Cardiovascular Diseases (I2MC), Team MetaDiab, Institut National de la Santé et de la Recherche Médicale (Inserm), Université Toulouse III, Paul Sabatier (UPS), UMR1297, Toulouse, France; 2 Nantes Université, CNRS, INSERM, Institut du Thorax, Nantes, France; 3 Nantes Université, CHU Nantes, Inserm, CNRS, SFR Santé, Inserm UMS 016, Nantes, France; 4 Mass Spectrometry Core Facility, CRNH-Ouest, Nantes, France; 5 Department of Human Biology, NUTRIM, School of Nutrition and Translational Research in Metabolism, Maastricht University Medical Centre+(MUMC+), Maastricht, The Netherlands; 6 Department of Clinical Biochemistry, Rigshospitalet, Copenhagen, Denmark; 7 Department of Biomedical Sciences, University of Copenhagen, Copenhagen, Denmark

**Keywords:** obesity, inflammation, lipoproteins, cytokines, adipokines, adipocytes, macrophages, sphingosine-1-phosphate, lipocalin

## Abstract

Obesity is associated with the development of local adipose tissue (AT) and systemic inflammation. Most adipokines are upregulated with obesity and have pro-inflammatory properties. Few are downregulated and possess beneficial anti-inflammatory effects. The apolipoprotein M (APOM) is an adipokine whose expression is low during obesity and associated with a metabolically healthy AT. Here, the role of adipose-derived APOM on obesity-associated AT inflammation was investigated by measuring the expression of pro-inflammatory genes in human and mouse models. In 300 individuals with obesity, AT *APOM* mRNA level was negatively associated with plasma hs-CRP. The inflammatory profile was assessed in *Apom*^−/−^ and WT mice fed a normal chow diet (NCD), or a high-fat diet (HFD) to induce AT inflammation. After HFD, mice had a higher inflammatory profile in AT and liver, and a 50% lower *Apom* gene expression compared with NCD-fed mice. *Apom* deficiency was associated with a higher inflammatory signature in AT compared with WT mice but not in the liver. Adeno-associated viruses encoding human *APOM* were used to induce *APOM* overexpression: in vivo, in WT mice AT prior to HFD; *in vitro*, in human adipocytes which conditioned media was applied to ThP-1 macrophages. The murine AT overexpressing *APOM* gene had a reduced inflammatory profile. The macrophages treated with APOM-enriched media from adipocytes exhibited lower *IL6* and *MCP1* gene expression compared with macrophages treated with control media, independently of S1P. Our study highlights the protective role of adipocyte APOM against obesity-induced AT inflammation.

Obesity is characterized by an excess of fat mass and is associated with a systemic and localized low-grade inflammation within the adipose tissue (AT) (1). The AT is not only a long-term energy storage organ but also secretes a large variety of bioactive factors, among which are adipokines. Adipokine secretion is altered with obesity and compelling evidence indicates that a variety of adipokines play an important role in obesity-related comorbidities (2). Most of the adipokines exhibit increased expression and secretion with obesity, and some act as pro-inflammatory factors to promote obesity-linked metabolic diseases (3). Conversely, few are down-regulated during obesity, and a defect in secretion of those anti-inflammatory adipokines (e.g., adiponectin), is likely to induce metabolic disturbances (4). Among those factors, we previously reported that the apolipoprotein M (APOM), which had been first discovered as produced by hepatocytes and kidney proximal tubule epithelial cells (5), then brain endothelial cells (6, 7), and in colorectal tissues (6, 7), is expressed and secreted by the adipocytes (8). Conflicting results exist regarding the link between plasma APOM and obesity, or type 2 diabetes (T2D). Plasma APOM is lower in individuals with obesity compared with lean patients (9), negatively associated with waist circumference (10) and lower in T2D compared with glucose-tolerant individuals (11, 12). However, a positive correlation between plasma APOM and leptin was reported (13). Mice models of T2D with hyperinsulinemia display lower plasma APOM levels compared with controls (14, 15), but streptozotocin-induced diabetes raised plasma APOM (16). The *APOM* gene expression in the AT is lower in individuals with metabolic syndrome, obesity, or T2D (8). The role of APOM in the AT remains unknown.

The human *APOM* gene is localized on chromosome 6p21.3 in the major histocompatibility complex of class III, which also includes genes of the TNF family. The APOM is a lipocalin, mainly known as a chaperone for sphingosine-1-phosphate (S1P), a sphingolipid with anti-inflammatory properties (17). In recent years, several evidence were provided about the protective effect of APOM, with S1P or not, against various inflammation processes (18). A negative association between plasma APOM and the C-reactive protein (CRP) has been previously reported (19, 20). We also reported a downregulation of AT *APOM* with obesity, metabolic syndrome, and T2D which are metabolic states associated with low-grade systemic and AT inflammation (8).

To the best of our knowledge, no data are currently available on the putative role of adipocyte-derived APOM within the AT. We, therefore, combined *in vivo* and *in vitro* experiments aiming to investigate the relationship between adipose APOM and AT inflammation. We compared AT inflammatory signature of *Apom*^*−/−*^ mice to those of WT littermates. The human *APOM* gene was specifically expressed in the perigonadal AT of WT mice. Further, the expression of *APOM* was forced in adipocytes, and conditioned media was used to culture macrophages before inducing pro-inflammatory phenotype.

The present study shows that APOM is one of these rare adipokines associated with a healthy metabolic phenotype that could be protective regarding AT inflammation and questions about the mechanism by which this may occur.

## Materials and methods

### Humans

Baseline AT samples and clinical data were obtained on 300 individuals (aged 42.5 ± 6.4 Y; 101 men and 199 women) with overweight or obesity (BMI: 34.1 ± 4.7 kg/m^2^) from the DiOGenes study (NCT00390637) (21). Abdominal subcutaneous AT biopsies were obtained by needle aspiration under local anesthesia after an overnight fast. The study was performed according to the latest version of the Declaration of Helsinki. Local ethics committees approved all procedures that involved human participants and written informed consent was obtained from all participants after verbal and written instructions.

### Animals

All animal experiments were approved by the Ethics Committee CEAA-122 and the Ministère de l’Enseignement Supérieur, de la Recherche et de l’Innovation and performed in the specific pathogen-free CREFRE animal facility, Inserm UMS006, University of Toulouse, National Veterinary School, France (DAP-APAFiS-2018111316011388). All animals were housed and manipulated according to the European Directive 2010/63/UE, and to the Inserm guidelines. All animals were housed in cages of 3 to 5 individuals, in a temperature-controlled environment with a 12-h dark/light cycle, in well-controlled animal care facilities. For the studies of weight gain and AAV transduction, 5-week-old C57BL/6J male mice were purchased from ENVIGO (Gannat, France).

For the study of APOM regulation following a weight gain, animals were fed either a normal chow diet (NCD, #EF D12450J, Ssniff) or a 60% high-fat diet (HFD, #EF D12492, Ssniff) at weaning time, and were housed either in a thermoneutral environment at 28–30°C in a vented animal cabinet or at standard temperature 22°C in the same room. Ten mice per group were killed after 1, 3, or 6 months of diet.

For the *APOM* gene overexpression study, the perigonadal fat pad of mice was transduced with adeno-associated viruses (AAVs) encoding either human APOM cDNA or *MCHERRY* cDNA, or injected with a physiological saline (Sigma-Aldrich). After surgery, mice were monitored daily and fed a 60% HFD for 5 weeks.

The *Apom*^−/−^ mice were provided by Christina Christoffersen from the University of Copenhagen, Denmark (22), and backcrossed with C57BL/6J mice to obtain heterozygous founders, which were intercrossed to get *Apom*^−/−^ mice and control *Apom*^+/+^ (WT) littermates. Sixteen male and female WT and *Apom*^−/−^ mice were housed in a thermoneutral environment at 28–30°C in a vented animal cabinet, other mice were housed in the same room at standard temperature (22°C). Body composition was assessed every 4 weeks with an NMR analyzer MiniSpec (Brucker Biospin). For the flow cytometry analysis of the perigonadal fat pad, 10 WT and 21 *Apom*^−/−^ female mice were housed at standard temperature and fed with either a 60% HFD or an NCD for 12 weeks.

At the end of all protocols, mice underwent a 3-h fasting before necropsies, and plasma was sampled for glucose measurement and further blood analysis. Tissues were immediately processed for flow cytometry analyses or flash frozen in liquid nitrogen and stored at −80°C until use for RNA isolation or protein extraction.

### AAVs

AAV vectors of serotype 8 encoding the human *APOM* cDNA, or *MCHERRY* cDNA as control were designed and produced by the Plateforme de Vectorologie of the Cancerology Research Center of Toulouse (Inserm UMR1037).

### Plasma measurements

Human high-sensitivity CRP (hs-CRP) was quantified by immunoturbidimetry using a Cobas Integra 400 (Roche Diagnostics). Murine CRP was dosed in plasma on a Luminex MAGPIX system (Luminex Corp) using a multiplex assay (MAP2MAG-76K, Merck Millipore).

### Flow cytometry analyses

Perigonadal fat pads were collected and processed as described in (23). Briefly, after weighing, the tissues were rinsed in cold Krebs-Ringer bicarbonate buffer (Sigma-Aldrich), minced then digested in PBS (Sigma-Aldrich) containing 2% bovine fatty acids free albumin (Sigma-Aldrich), 1% type I collagenase (Sigma-Aldrich), 60 μg/ml liberase (Roche), 250 IU/ml hyaluronidase (Sigma-Aldrich) for 1h at 37°C under agitation. Digested tissues were filtrated through 225 μm pore filters and centrifuged at 1,500 rpm for 5 min. After washing with PBS, the adipocytes-containing supernatants were discarded and the pelleted stromal vascular fractions (SVF) were suspended in erythrocyte lysis buffer (8 g/L NH_4_Cl, 1 g/L K_2_HPO_4_, 0.04 g/L EDTA) and incubated for 10 min at room temperature with constant agitation. The SVF cells were then centrifuged at 1,500 rpm for 5 min and 0.5∗10^6^ cells were suspended in PBS supplemented with 0.5% albumin and 2 mM EDTA (Sigma-Aldrich). Live cells were stained for 20 min using a LIVE/DEAD Fixable blue stain (Invitrogen) diluted 1/1,000 in PBS. Pro-inflammatory macrophages were stained using anti-CD45-PE-Vio 770 REAfinity (clone REA737, Miltenyi, Biotec), anti-F4/80-APC (clone BM8, BioLegend), anti-CD11b-PE-Vio 615 REAfinity (clone REA592, Miltenyi) and anti-CD11c-Brilliant Violet 711 (clone N418, BioLegend) antibodies. Cells were analyzed using an Aurora Cell Sorter flow cytometer (Cytek Biosciences). Results were acquired using the OMIQ software (OMIQ).

### Histological analyses

Perigonadal AT from WT and *Apom*^−/−^ mice fed a 60% HFD for 12 weeks were fixed with 4% paraformaldehyde (Sigma-Aldrich) in PBS, dehydrated, embedded in paraffin, and cut into 5 μm sections. Sections were stained with hematoxylin and eosin using standard protocols. Digital images of the entire fat pad sections were captured using a light microscope coupled to a camera (NanoZoomer 2.0 RS Hamamatsu) and analyzed using the morphometric program Fiji v2.1.0 (ImageJ, NIH) with the adipocytes automated detection plugin AdipoTool. The crown-like structures (CLS) in the whole fat pad sections were counted blindly by two analysts. The CLS density was expressed per mm^2^ of tissue section.

### Cell culture

To mimic in vitro the human AT landscape, cell lines were used instead of primary cultured cells to ensure concomitant availability of each cell type.

Human multipotent adipose-derived stem (hMADS) cells were cultured as previously described (8). Cells were differentiated in serum-free DMEM low-glucose/Ham’s F-12 (Lonza, Basel, Switzerland) medium supplemented with 125 nM transferrin, 10 nM insulin, 0.2 nM triiodothyronine, 100 μM 3-isobutyl-1-methylxanthine (IBMX), 1 μM dexamethasone (Sigma-Aldrich) and 100 nM rosiglitazone (Cayman Chemical). After 6 days of differentiation (day 6), dexamethasone and IBMX were omitted from the medium. Rosiglitazone was omitted at day 9 and on day 13, hMADS cells were fully differentiated.

Human ThP-1 monocytes were cultured in suspension in RPMI-1640 medium containing 10% FBS, 1 mM sodium pyruvate, 50 μM 2-mercaptoethanol (Invitrogen), and 36 mM glucose (Sigma-Aldrich). For differentiation, cells were seeded in a serum-free, 2-mercaptoethanol-free RPMI medium containing 100 nM phorbol 12-myristate 13-acetate (PMA, Sigma-Aldrich) for 48 h. Pro-inflammatory macrophage polarization was triggered with 2 ng/ml *Escherichia coli* lipopolysaccharide (LPS, Sigma-Aldrich) and 10 ng/ml IFNγ (Sigma-Aldrich). Cells were fully polarized after 3 days.

### Treatments

For the study of APOM regulation, hMADS were treated at day 10 with 10 or 50 μg/ml CRP (Merck Millipore) for 48 h. For AAV transduction, the hMADS adipocytes were transduced at day 10 as described in (24). On day 13, conditioned media were sampled. ThP-1 cells were treated with 50/50 conditioned media from transduced hMADS cells and RPMI, with 1 μM S1P prepared in 0.4% BSA when indicated, one day prior to polarization (25). All cells were lysed using RLT buffer (Qiagen, GmbH) containing 10% 2-mercaptoethanol (Sigma-Aldrich), flash frozen, and stored at −80°C until use.

### Human APOM measurements

Human APOM was dosed in hMADS-conditioned media as described in (26). Briefly, medium samples were concentrated in ammonium bicarbonate buffer with a 5 kDa molecular mass cut-off filter. Concentrated samples were then reduced, alkylated and trypsin digested overnight. Human APOM was quantified using proteotypic signature peptide AFLLTPR and labeled synthetic peptide (AFLLTP-[^13^C_6_,^15^N_4_]R) as an internal standard. Labeled and unlabeled peptides were then separated and detected by liquid chromatography-mass spectrometry (LC-MS/MS) as described previously (26). Data acquisition and analyses were performed with MassLynx® and TargetLynx® software, respectively (Waters Corporation).

### RNA extraction and RT-PCR

Tissues were lysed in Qiazol reagent (Qiagen) and RNA was extracted using an RNeasy mini kit (Qiagen) following the manufacturer’s instructions. Total RNA (500 ng) was reverse transcribed for 120 min at 37°C using Superscript II reverse transcriptase (Invitrogen) in the presence of random hexamers. Real-time PCR was performed using Taqman probes or Sybergreen primers assays (Table S1) with a QuantStudio 5 Real-Time PCR system (Applied Biosystems). Expression of murine *Tnfa*, Interleukin 1b *(Il1b),* Interleukin 6 *(Il6),* Monocyte chemoattractant protein 1 *(Mcp1),* Cluster of differentiation 68 (*Cd68*), Cluster of differentiation 11c (*Cd11c*), Serum amyloid A3 (*Saa3*) and *Apom* was measured in all murine tissues; expression of human *APOM* and *MCHERRY* gene were additionally measured in AT from the *APOM* gene overexpression study. Expression of the human *APOM* gene was measured in AT of human subjects and human *TNFA*, *IL1B*, *IL6*, *MCP1*, *APOM,* and *MCHERRY* were measured in in vitro models. The murine tissue data were normalized to the mean between Hypoxanthine phosphoribosyltransferase *(Hprt)* and TATA-binding protein (*Tbp*). The human tissue data were normalized to Pumilio RNA-binding family member 1 (*PUM1*). The hMADS and ThP-1 data were normalized to LDL receptor-related protein 10 (*LRP10*) and Proteasome 26S subunit, ATPase 4 (*PSMC4*), respectively.

### Western blot

AT proteins were extracted using a RIPA buffer (Sigma-Aldrich) and quantified using a Pierce BCA assay kit (ThermoFisher). Proteins were separated using a 4%–20% SDS-PAGE. Gels and nitrocellulose membranes were exposed to UV light in a ChemiDoc imaging system (Bio-Rad) to permit Stain-Free normalization. Membranes were incubated overnight with primary antibodies anti-APOM (H00055937-M03, Abnova, Taipei, Taiwan) 1/1,000, or anti-β-actin (#4970, Cell Signalling Technology,) 1/2000, diluted in TBS-T (10 mM Tris, 150 mM NaCl, 0.2% Tween-20) buffer containing 5% skimmed milk. After washing, membranes were incubated with HRP-conjugated secondary antibodies, and proteins were visualized using a Clarity ECL reagent and ChemiDoc imaging system (Bio-Rad). Data were analyzed using Image Lab™ software (Bio-Rad) version 6.1. Signal intensity was normalized to the total protein signal, using the Stain-Free Technology, in each lane.

### Statistical analysis

Statistical analyses were performed using GraphPad Prism software v9.4.1 (GraphPad Software Inc) or R software v4.0.5 with the factoextra and FactoMineR packages. The centroid of mean gene expression was calculated as the mean of geometric means of all gene's mRNA levels as described in (27, 28, 29) to represent a weighted average of several gene expression levels. Spearman or Pearson correlation was applied when appropriate. Mann-Whitney tests were performed to determine differences between groups followed by the Benjamini-Hochberg procedure to control for multiple comparisons when appropriate. Kruskal-Wallis or two-way ANOVA followed by Sidak post hoc test were applied when appropriate. All values in the figures are presented as mean ± SEM. Statistical significance was set at *P* < 0.05.

## Results

### At *APOM* gene expression is negatively associated with systemic and inflammation

**Fig. 1:**
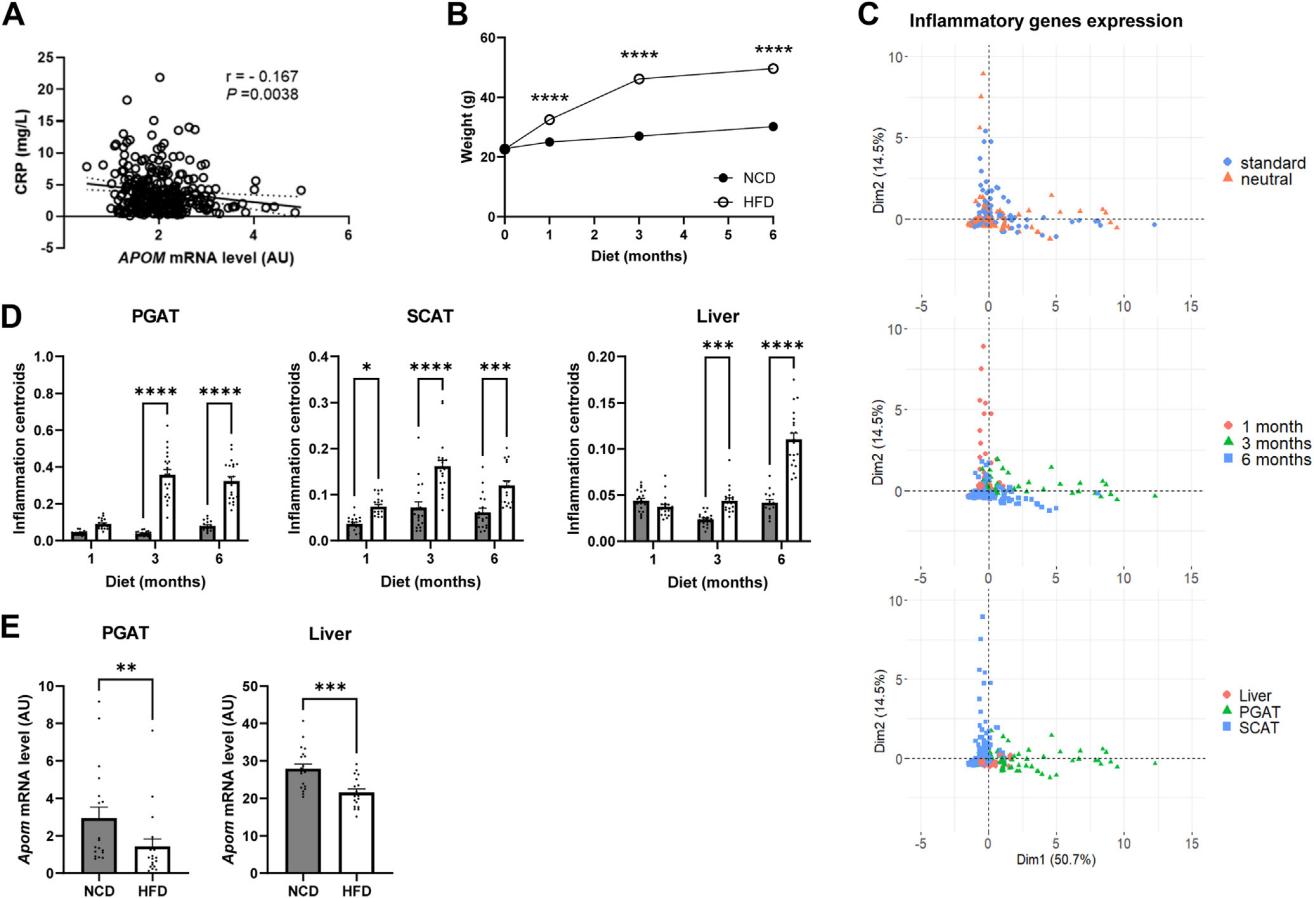
Association between weight gain-induced inflammation and APOM expression in AT and liver in humans and mice. A: Correlation between plasma hs-CRP and *APOM* gene expression in abdominal SCAT from subjects with obesity or overweight (n = 300). Pearson correlation coefficient is presented. Dotted lines represent 95% confidence interval. B: Mice body weight after a NCD or a HFD for 1, 3 or 6 months (n = 10 per group). White dots represent HFD-fed mice and black dots NCD-fed mice. C: Principal component analysis of inflammatory gene expression in HFD-fed mice according to housing temperature, diet duration and tissue type (n = 10 per group). D: Centroids of inflammatory genes expression in perigonadal, subcutaneous AT and liver of mice housed at standard and neutral temperature, after 1, 3 or 6 months of NCD or HFD (n = 10 per group). Data were analyzed by Kruskal-Wallis with Sidak post-hoc test. E: *Apom* expression in perigonadal AT and liver of mice housed at standard and neutral temperature, after 3 months of NCD or HFD (n = 10 per group). Results were analyzed by Mann–Whitney’s *U* test between NCD and HFD groups at each time. Results are presented as mean ± SEM. ∗, *P* < 0.05, ∗∗, *P* < 0.01, ∗∗∗, *P* < 0.001, ∗∗∗∗, *P* < 0.0001. AU, arbitrary unit; hs-CRP, high sensitive C-reactive protein; HFD, high-fat diet; NCD, normal chow diet; PGAT, perigonadal adipose tissue; SCAT, subcutaneous adipose tissue.

As a negative association between the circulating CRP and APOM was repeatedly reported in humans (19, 30), we questioned the relationship between *APOM* gene expression in human subcutaneous AT and circulating hs-CRP in 300 individuals with overweight or obesity (supplemental Table S2). An inverse association between adipose *APOM* mRNA level and hs-CRP (*r* = −0.167; *P* = 0.0038) was found (Fig. 1A).

To investigate whether weight gain could regulate *Apom* expression, mice were fed with an HFD or NCD for 1, 3, and 6 months. Weight gain of HFD-fed mice reached a plateau at 3 months of diet (Fig. 1B). Since housing temperature can alter the inflammatory response to HFD (31, 32) and metabolic phenotype (33), mice were housed either at 22°C or 30°C. We measured the expression of 7 genes encoding inflammatory proteins, namely *Tnfa*, *Il1b*, *Il6*, *Mcp1*, *Cd68*, *Cd11c,* and *Saa3*, in perigonadal AT (PGAT), inguinal subcutaneous AT (SCAT) and in the liver. Principal component analysis (PCA) was performed based on this inflammatory gene expression panel (Fig. 1C). PGAT, SCAT, and liver were distinctly separated by the two principal components. No effect of housing temperature was observed. Therefore, we chose to analyze data independently of housing temperature. Figure 1D shows the centroids calculated on the inflammatory gene expression in PGAT, SCAT, and liver. Inflammatory signature was higher after 3 and 6 months of HFD in both fat pads and liver. Therefore, we chose to analyze PGAT and liver data, at 3 months of HFD. Figure 1E shows that the *Apom* mRNA level in PGAT and liver was significantly decreased after a 3-month HFD.

### The *Apom* deficiency is associated with higher systemic and inflammation

**Fig. 2:**
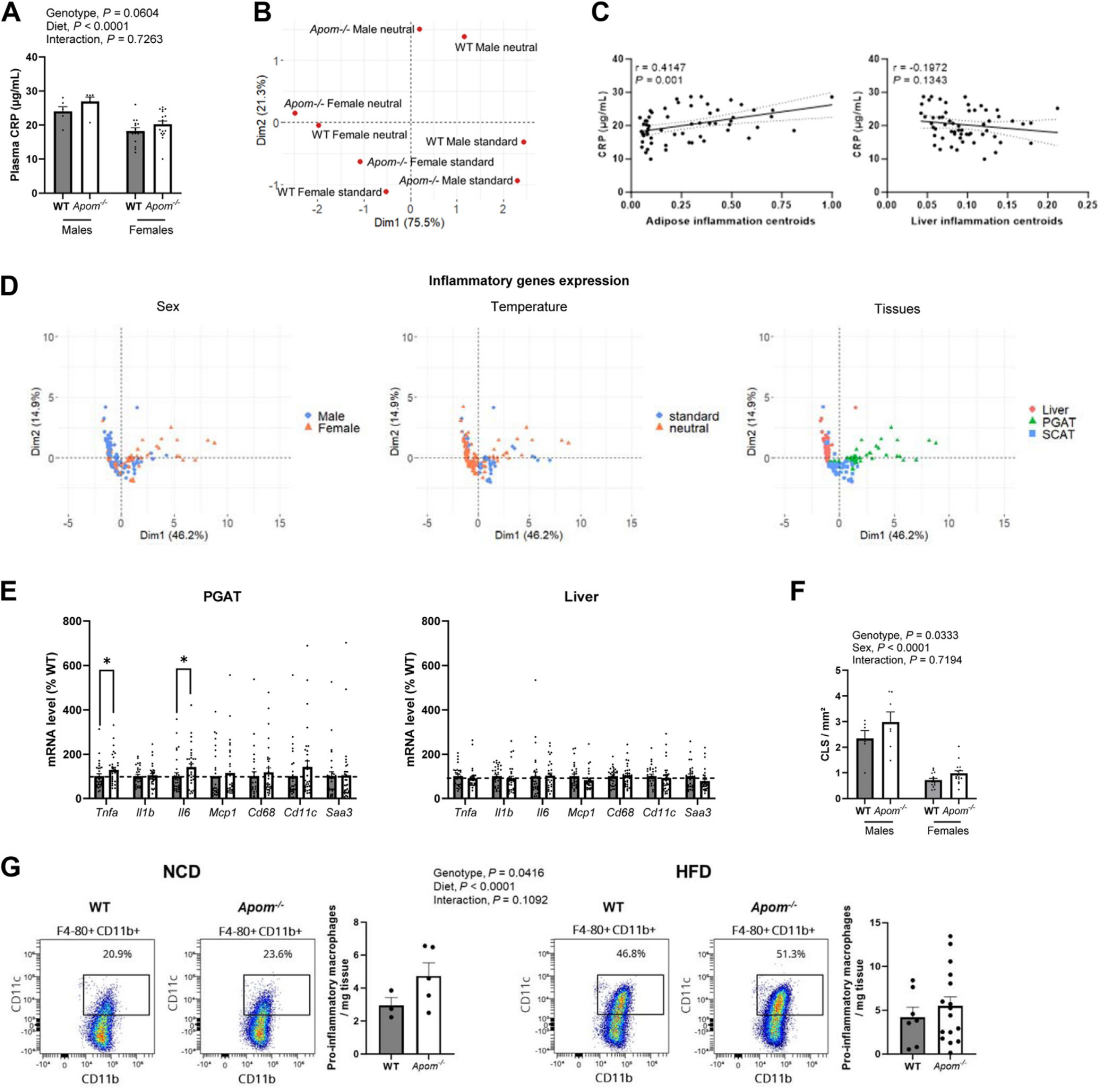
Effect of *Apom* deficiency on AT inflammation. Mice were fed a HFD for 3 months and were housed at standard or neutral temperature. A: Plasma CRP dosage in mice housed at standard temperature (n = 6–17 per group). Dark bars, WT mice; white bars, *Apom*^*−/−*^ mice. Results were analyzed by Kruskal-Wallis with Sidak post-hoc test. B: Principal component analysis of phenotypic data centroids according to genotype, sex and housing temperature. C: Correlation between plasma CRP and centroids calculated on inflammatory gene expression in PGAT or liver of WT and *Apom*^*−/−*^ mice (n = 60). Spearman correlation coefficient is presented. Dotted lines represent 95% confidence interval. D: Principal component analysis of inflammatory genes expression according to sex, housing temperature and tissue type. E: Inflammatory genes expression in PGAT and liver of mice housed at standard and neutral temperature (n = 29–32). Dark bars, WT mice; white bars, *Apom*^*−/−*^ mice. Data are expressed as percentage of WT mice and were analyzed by Mann–Whitney *U* test followed by Benjamini-Hochberg procedure. Results are presented as mean ± SEM. F: Number of CLS per mm^2^ of PGAT in WT and *Apom*^*−/−*^ mice fed an HFD for 3 months (n = 6–17 per group). Results were analyzed by two-way ANOVA with Sidak post-hoc test. G: Flow cytometry analyses performed on PGAT of WT (n = 10) and *Apom*^*−/−*^ (n = 21) female mice fed an HFD (n = 23) or NCD (n = 8) for 3 months. The pro-inflammatory macrophages (CD45+ F4/80+ CD11b + CD11c+) absolute number is expressed per milligram of PGAT. Data were analyzed by two-way ANOVA with Sidak post-hoc test. Results are presented as mean ± SEM. ∗, *P* < 0.05. AT, adipose tissue; CLS, crown-like structures; CRP, C-reactive protein; HFD, high-fat diet; NCD, normal chow diet; PGAT, perigonadal adipose tissue; SCAT, subcutaneous adipose tissue.

To study the role of APOM in the AT, we investigated the effect of *Apom* deficiency on HFD-induced AT inflammation in *Apom*^*−/−*^ and WT mice fed an HFD for 3 months. Body weight and composition, as well as adipocyte sizes in PGAT, were similar between *Apom*^*−/−*^ and WT mice both before and after HFD (supplemental Figs. S1 and S2). After the 3-month HFD, plasma CRP was higher in males compared with females (*P* < 0.0001) and exhibited a trend (*P* = 0.0604) to be higher in *Apom*^*−/−*^ compared with WT mice (Fig. 2A). Figure 2B displays the PCA of the body composition of mice according to sex, genotype, and housing temperature. Components 1 and 2 explained 96.8% of data variability. No impact of genotype or housing temperature was observed.

We then examined the gene expression of the 7 pro-inflammatory markers in PGAT. For each tissue/group of mice, the centroid of mean gene expression was calculated (28). A positive association between plasma CRP and inflammation centroids in AT was found (*r* = 0.415, *P* = 0.001), but not in the liver (Fig. 2C). Figure 2D shows the PCA based on the expression of the genes encoding inflammatory markers. The maximal amount of variance came from tissues, whereas sex or housing temperature had no effect. We therefore examined gene expression data independently of sex and housing temperature. Figure 2E shows the gene expression of each marker. Compared with WT mice, a higher expression in *Apom*^*−/−*^ mice was found for *Tnfa* and *Il6* (*P <* 0.05), in the PGAT, not in the liver. Also, the inflammation centroids mean in PGAT were 0.290 (CI_95_ = [0.212;0.368]) for *Apom*^*−/−*^ mice and 0.258 for WT mice (CI_95_ = [0.178;0.338]), and 0.113 (CI_95_ = [0.067;0.159]) for *Apom*^*−/−*^ mice and 0.102 (CI_95_ = [0.088;0.115]) for WT mice in liver (data not shown). Altogether, this indicates that the PGAT of *Apom*^*−/−*^ mice under HFD display a more pronounced inflammatory signature than the PGAT of the WT littermates.

In the AT, after a 3-month HFD, the density of CLS was 1.44-fold (*P* = 0.033) higher in *Apom*^*−/−*^ than in WT mice (supplemental Fig. S3 and Fig. 2F). The PGAT immune cells content was analyzed by flow cytometry in *Apom*^*−/−*^ and WT mice after 3 months of HFD, or NCD. Pro-inflammatory F4/80^+^ CD11b^+^ CD11c^+^ macrophages number was studied following the gating process described in supplemental Fig. S4. Figure 2G shows the number of F4/80^+^ CD11b^+^ CD11c^+^ (pro-inflammatory) macrophages per mg of PGAT. As expected, HFD-fed mice had a higher proportion of pro-inflammatory macrophages among total macrophages compared with NCD-fed mice (*P* < 0.0001). The *Apom*^*−/−*^ mice displayed a higher number of F4/80^+^ CD11b^+^ CD11c^+^ cells among total macrophages than WT mice (*P* = 0.0416). No significant interaction was found between diet and genotype (*P* > 0.1).

### Adipose tissue *APOM* overexpression is associated with lower local inflammation

**Fig. 3:**
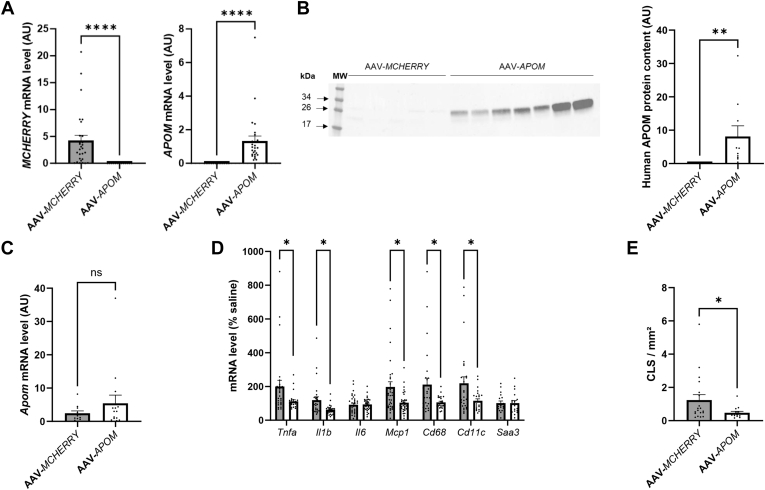
Effect of *APOM* overexpression on AT inflammation. PGAT of mice were transduced with AAVs encoding MCHERRY (AAV-*MCHERRY*) or human APOM (AAV-*APOM*), and the mice were fed a HFD for 5 weeks (n = 26–28 per group). A: Expression of *MCHERRY* and *APOM* in PGAT. B: Human APOM protein level in PGAT. A representative Western blot with 30 μg in each lane and a 65 s exposure is shown. Results are expressed as a percentage of the total protein (AAV-*MCHERRY,* n = 28; AAV-*APOM*, n = 26). Data were analyzed by Mann–Whitney *U* test. C: Expression of murine gene *Apom* in PGAT (n = 26–28 per group). Data were analyzed by Mann–Whitney *U* test. D: Inflammatory genes expression in PGAT of mice are expressed as percentage of saline group (n = 26–28 per group). Data were analyzed by the Mann–Whitney *U* test followed by Benjamini-Hochberg adjustment. E: Number of CLS per mm^2^ of PGAT (n = 26–28 per group). Data were analyzed by Mann–Whitney *U* test. In gray, AAV-*MCHERRY* transduction; in white, AAV-*APOM* transduction. Results are presented as mean ± SEM. ∗, *P* < 0.05, ∗∗, *P* < 0.01, ∗∗∗∗, *P* < 0.0001. AAV, adeno-associated virus; AT, adipose tissue; AU, arbitrary units; CLS, crown-like structures; HFD, high-fat diet; PGAT, perigonadal adipose tissue.

We tested *in vivo* whether overexpressing *APOM* while promoting obesity could limit obesity-induced inflammation development in AT. The PGAT of WT mice was transduced with AAV encoding either the human *APOM* cDNA or the control cDNA, *MCHERRY*. Animals were subsequently fed an HFD for 5 weeks to trigger the development of inflammation in AT (Fig. 3). As expected, *MCHERRY* or *APOM* gene expression were only found in the PGAT of mice transduced with the AAV-*MCHERRY* or AAV-*APOM*, respectively (Fig. 3A), and the human APOM protein was only detected in the latter (Fig. 3B and supplemental Fig. S5). No difference in the murine *Apom* gene expression between the groups (*P* = 0.7496) was found (Fig. 3C). Figure 3D shows the expression of the pro-inflammatory markers in PGAT after HFD. Expression of inflammatory genes was lower in the PGAT of mice transduced with AAV-*APOM* compared with PGAT of mice transduced with AAV-*MCHERRY*. The inflammation centroids means were 90.49 (CI_95_ = [80.0; 101.0]) for AAV-*APOM* transduced fat pad and 137.31 (CI_95_ = [102.8; 171.8]) for AAV-*MCHERRY* transduced fat pad. The density of CLS was 62% lower (*P* = 0.034) in the PGAT of mice transduced with the AAV-*APOM* than in the PGAT of mice transduced with the AAV-*MCHERRY* (Fig. 3E).

### *APOM* overexpression in adipocytes restrains macrophages pro-inflammatory polarization

**Fig. 4:**
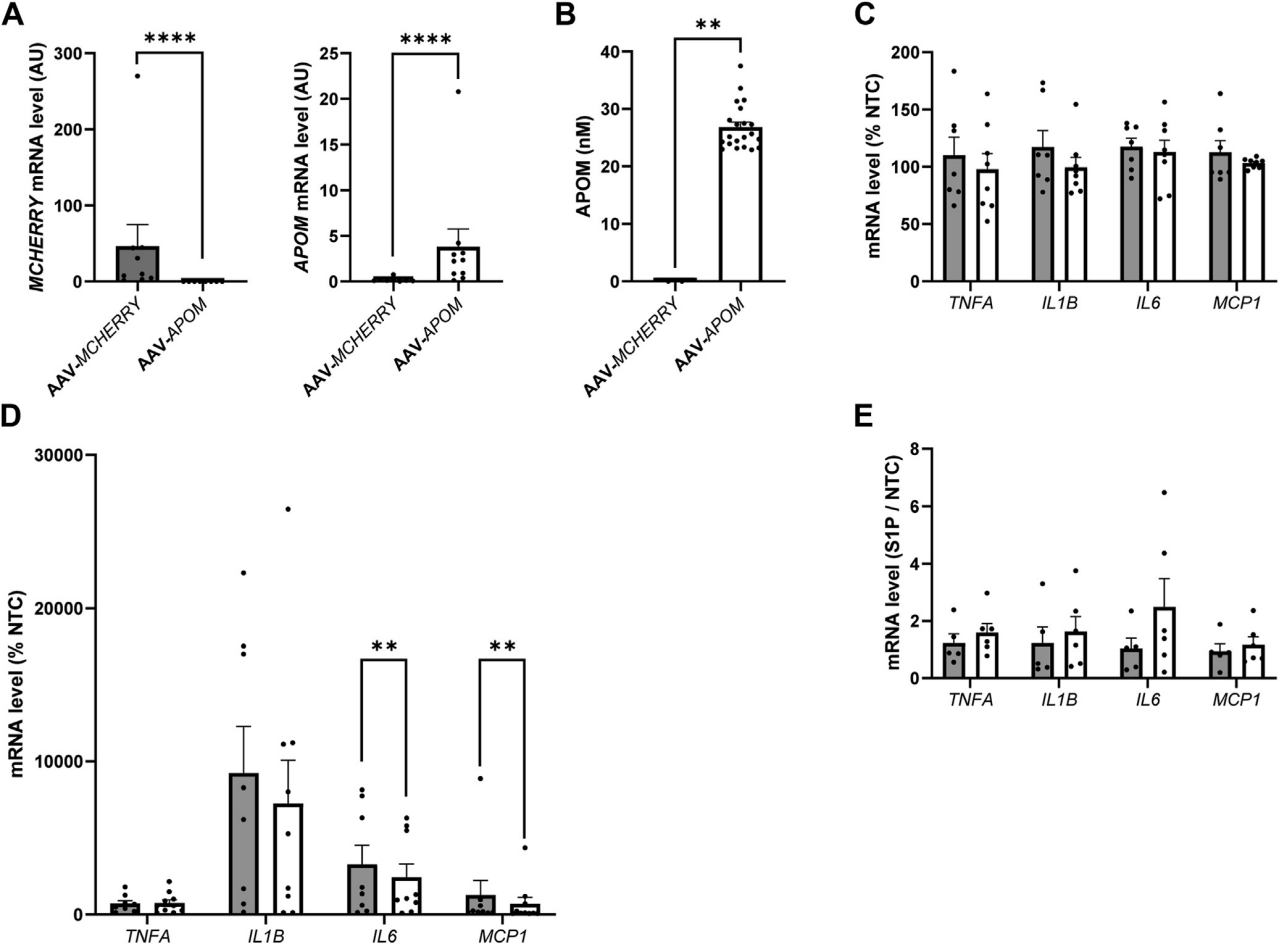
Effect of *APOM* overexpression on adipocytes and macrophages. hMADS cells were transduced with AAVs encoding APOM (AAV-*APOM*) or MCHERRY (AAV-*MCHERRY*), then ThP-1 cells were treated with conditioned media from hMADS cells before polarization (n = 9). A: Expression of *MCHERRY* and *APOM* in AAV-transduced hMADS cells (n = 8–10 per condition). Data were analyzed by Mann–Whitney *U* test. B: APOM concentration in conditioned media from AAV-transduced hMADS cells. Data were analyzed by Mann–Whitney *U* test. C: Inflammatory cytokines expression in transduced hMADS cells expressed as percentage of non-transduced cells. Data were analyzed by Mann–Whitney *U* test followed by Benjamini-Hochberg adjustment. Grey bars represent AAV-*MCHERRY* transduced hMADS cells, and white bars represent AAV-*APOM* transduced hMADS cells. D: Inflammatory cytokines expression in pro-inflammatory polarized ThP-1 cells with conditioned media from hMADS cells (n = 9). Data are expressed as a percentage of non-polarized ThP-1. Data were analyzed by Mann–Whitney *U* test followed by Benjamini-Hochberg adjustment. E: Ratios of inflammatory cytokines expression in ThP-1 with conditioned media from hMADS cells and S1P, on gene expression in ThP-1 without S1P (n = 5–6 per condition). Data were analyzed by Mann–Whitney *U* test followed by Benjamini-Hochberg adjustment. Gray bars represent conditioned media from hMADS cells transduced with AAV-*MCHERRY;* white bars represent conditioned media from hMADS cells transduced with AAV-*APOM*. Results are presented as mean ± SEM. ∗∗, *P* < 0.01, ∗∗∗∗, *P* < 0.0001. AAV, adeno-associated virus; AU, arbitrary unit; NTC, non-transduced cells; S1P, sphingosine-1-phosphate.

To mimic in vitro the effect of adipocyte-derived APOM on macrophage polarization in AT, hMADS adipocytes were transduced with either AAV-*MCHERRY* or AAV-*APOM* (Fig. 4). Both AAV-*MCHERRY* and AAV-*APOM* promoted *MCHERRY* and *APOM* gene expression (Fig. 4A) and the human APOM protein was found in media from AAV-*APOM* transduced cells at a mean concentration of 26.81 ± 0.87 nM (Fig. 4B). Figure 4C displays the mRNA levels of 4 cytokines which are secreted by adipocytes (34, 35). Cytokine gene expression in hMADS adipocytes was similar between AAV-*MCHERRY* and AAV-*APOM* transduced cells.

The conditioned media from hMADS cells were used to treat non-polarized (M0) ThP-1 cells. The cells were thereafter polarized into pro-inflammatory macrophages. *IL6* and *MCP1* gene expression was lower (*P* < 0.01) in pro-inflammatory macrophages treated with APOM-enriched media compared with control (MCHERRY) media (Fig. 4D).

As a lipocalin, APOM binds S1P, which has anti-inflammatory properties (17). We thus tested if the effect of APOM-enriched media on macrophages was S1P-dependent. We compared the effect of the conditioned media from hMADS, with or without S1P supplementation, on the pro-inflammatory polarization of ThP-1 cells. Figure 4E shows the ratio of cytokine gene expression in ThP-1 cells treated with conditioned media in the presence of S1P normalized to the expression level in ThP-1 cells treated with conditioned media without S1P. No difference was found, suggesting that the effect of APOM on macrophages was independent of S1P.

## Discussion

In recent years, the APOM has gained interest as a novel player of inflammation. This small lipocalin binds several small lipidic molecules, especially S1P, and the APOM/S1P complex displays anti-inflammatory properties (36). As circulating APOM is negatively associated to plasma CRP in various inflammatory conditions, some authors have qualified the APOM as a negative acute phase protein (19, 30). In addition, several studies have provided evidence regarding the beneficial role of APOM, with its main ligand S1P, in severe inflammatory contexts, including sepsis (37), heart failure (38), auto-immune diseases (20) and cancers (39). However, the role of APOM in a low-grade inflammatory context, such as obesity-associated inflammation, has not been studied yet. The development of obesity is characterized by AT expansion, which triggers anatomical and functional alterations such as hypertrophy and hyperplasia, tissue fibrosis, enhanced deleterious adipokines secretion, and immune cell recruitment (40). Overall, obesity is associated with a low-grade inflammatory state both at the systemic and tissue levels (1). We previously identified APOM as an adipokine whose gene expression in AT is lower in individuals with obesity, metabolic syndrome, or T2D, compared with healthy ones (8).

In this study, we examined the adipocyte APOM with regard to the inflammatory status. First, the *APOM* gene expression in AT was negatively correlated to plasma CRP in individuals with overweight or obesity, indicating that adipose APOM is inversely associated with obesity-related low-grade systemic inflammation.

To study the regulation of the adipose APOM in an inflammatory context, WT mice were exposed to HFD. An HFD increases circulating CRP (41) and induces a shift in bacterial species in the gut microbiota, with an increase in lipopolysaccharide-secreting species and consequent activation of the inflammasome (42). Several saturated fatty acids, such as lauric acid or palmitic acid, have been identified as pro-inflammatory fatty acids because they are recognized by Toll-like receptor 4, which leads to the transcription of inflammasome components (43). In the AT, the HFD-induced weight gain is responsible for an unbalanced secretion of adipokines which favors pro-inflammatory cytokines and a recruitment of Th17 lymphocytes and dendritic cells which promote M1-like polarization of macrophages (44). We found that the HFD downregulates *Apom* gene expression in AT and liver, suggesting that APOM is negatively regulated by obesity-associated inflammation. This observation is supported by our previous findings indicating a negative influence of TNFA, or CRP, on *APOM* gene expression in human adipocytes (8, 45), as well as in a study from Gao *et al.* in human hepatocytes (46).

Second, we investigated the systemic and local inflammation profile in AT of *Apom*^*−/−*^ mice. We previously reported a negative association between AT *APOM* mRNA level and adipocyte size in women with obesity (8). In the present mouse models, no difference in body weight or composition, nor in adipocyte size, was found between *Apom*^*−/−*^ and WT mice. However, we found a higher number of pro-inflammatory macrophages and CLS in AT of *Apom*^*−/−*^ mice. This observation suggests that *Apom* deficiency might promote the mobilization of monocyte-derived pro-inflammatory macrophages to the AT. We also found a higher inflammatory signature in AT of *Apom*^*−/−*^ mice. This was not detected in the liver. Also, surprisingly, we found a positive association between plasma CRP and inflammatory gene expression only in the AT, but not in the liver. The discrepancies between those tissues may suggest that the *Apom* deficiency is more deleterious for AT than for the liver, in the context of obesity-induced inflammation, but such a difference could be because the genes investigated here are not the most representative of liver inflammation.

Because housing temperature may impact inflammation (31), mice were housed at standard and neutral temperatures. Interestingly, we did not find any difference in the AT inflammatory gene expression profile between standard-housed and neutral-housed mice. The impact of ambient temperature during housing on body composition and inflammation is still a debated question: although several authors reported increased body weight, adipose mass, adipocyte size (47) and plasma pro-inflammatory cytokines levels in neutral-housed compared with standard-housed mice (31), other studies showed a similar adipose mass (48) and little differences in plasma cytokines levels between the two groups of mice (47). Also, housing temperature was reported to impact body temperature in *Apom*^*−/−*^ mice compared with WT mice when housed at standard but not at neutral temperature (49). In the liver, thermoneutral housing induces an increased collagen deposition and fibrosis as well as an increased infiltration of lymphocytes and innate immune cells (50), but not macrophages (51). Almost all the markers that we measured are linked to AT macrophages and therefore do not exactly recapitulate liver inflammation. Our observation is consistent with the study of Tian *et al.*, in which macrophage count was similar in AT from standard-housed and neutral-housed mice after 12 weeks of HFD, despite an earlier infiltration of these cells in AT from neutral-housed mice (48).

Then we aimed to explore the role of the adipose-derived APOM on AT during an obesity-induced inflammation. To simulate the effect of adipose APOM in a healthy context, we forced human *APOM* genes specifically in the PGAT of mice and in human adipocytes using AAVs. We found that human APOM locally prevents the obesity-associated inflammation of the AT. The APOM may promote the impediment of macrophage inflammatory polarization and limit the ability of activated macrophages to recruit circulating monocytes, through a lower MCP1 expression. Moreover, in our study, this novel role for APOM in AT may be independent of S1P. This observation was unexpected because no specific receptor for APOM has been identified on mature adipocytes and the APOM effect is likely mediated by its ligands. Abboud and colleagues have reported the expression of the megalin receptor only on pre-adipocytes, which then decreased during the differentiation of the adipocytes (52). Of note, we were unable to reveal gene expression of the megalin receptor in the hMADS adipocytes (data not shown). Although S1P is the most studied ligand for APOM, this lipocalin can bind other lipidic molecules including retinoic acid, oxidized phospholipids and C14 to C18 fatty acids (53, 54). Retinol as well as polyunsaturated fatty acids have anti-inflammatory properties, as reviewed in (55, 56). Therefore, we cannot exclude a local anti-inflammatory effect of APOM with these lipid species. Likewise, Yore *et al.* have identified hydroxylated fatty acids as promising anti-inflammatory lipids (57). Whether APOM binds any of those branched-chain fatty acids deserves further interest. Also, whether the adipose APOM is similar to that produced by the liver, and so binds S1P, is an open question.

This study has several limitations, some of them being due to the use of a murine model. Indeed, advances in the understanding of mice physiology have led to reconsidering animal feeding, breeding, and housing strategies, in addition to individual-linked variables (age, sex, and genetic background among others). Also, mice have higher HDL particles than LDL particles, whereas humans have the opposite, but plasma lipidomics studies showed strong correlations between humans and mice (r = 0.810, *P* < 0.0001) unlike the other species such as dogs and rats (58). The APOM is mainly bound to HDL particles (59), suggesting that variations of plasma lipids contents can also modulate plasma APOM levels in mice. In studying lipoproteins components, one has to consider using pigs or guinea pigs to extrapolate the data to humans or using isolated lipoproteins from human donors. Nevertheless, mouse models have been extensively used to study APOM (49, 60, 61). In addition, despite we previously reported that the AT releases more APOM after a caloric restriction (8), we must acknowledge that the overexpression experiments do not mimic what happens in a physiological context. Also, as the adipocytes may account for less than half of the AT cells, notably in individuals with obesity (62), our in vitro experiments do not fully recapitulate physiology. Lastly, the murine APOM protein, despite a sequence homology of 81% with the human APOM, has a different lipocalin fold than the human APOM. Lipocalins are typically formed by a β-barrel composed of 8 strands, while the murine APOM only has 7 strands (63). Whether this unusual conformation has an impact on APOM binding properties, particularly regarding its anti-inflammatory ligands, is an unaddressed question. Therefore, the use of a humanized model, such as the present AAV-induced model of human *APOM* gene overexpression, and better, in species that exhibit a similar lipoprotein profile than that of humans, deserves consideration.

Overall, our study highlights the relationship between APOM and obesity-associated low-grade inflammation and proposes adipose-derived APOM as a novel and local protective factor against AT inflammation.

## Data availability

Data are available on reasonable request.

## Supplemental data

This article contains supplemental data.

## Conflict of interests

The authors declare that they have no conflicts of interest with the contents of this article.

## References

[1] Ouchi N., Parker J.L., Lugus J.J., Walsh K. (2011). Adipokines in inflammation and metabolic disease. Nat. Rev. Immunol..

[2] Fasshauer M., Bluher M. (2015). Adipokines in health and disease. Trends Pharmacol. Sci..

[3] Scheja L., Heeren J. (2019). The endocrine function of adipose tissues in health and cardiometabolic disease. Nat. Rev. Endocrinol..

[4] Maeda N., Funahashi T., Matsuzawa Y., Shimomura I. (2020). Adiponectin, a unique adipocyte-derived factor beyond hormones. Atherosclerosis.

[5] Dahlback B., Nielsen L.B. (2009). Apolipoprotein M affecting lipid metabolism or just catching a ride with lipoproteins in the circulation?. Cell Mol. Life Sci..

[6] Luo G., Zhang X., Mu Q., Chen L., Zheng L., Wei J. (2010). Expression and localization of apolipoprotein M in human colorectal tissues. Lipids Health Dis..

[7] Kober A.C., Manavalan A.P.C., Tam-Amersdorfer C., Holmer A., Saeed A., Fanaee-Danesh E. (2017). Implications of cerebrovascular ATP-binding cassette transporter G1 (ABCG1) and apolipoprotein M in cholesterol transport at the blood-brain barrier. Biochim. Biophys. Acta Mol. Cell Biol Lipids.

[8] Sramkova V., Berend S., Siklova M., Caspar-Bauguil S., Carayol J., Bonnel S. (2019). Apolipoprotein M: a novel adipokine decreasing with obesity and upregulated by calorie restriction. Am. J. Clin. Nutr..

[9] Ooi E.M., Watts G.F., Chan D.C., Nielsen L.B., Plomgaard P., Dahlback B. (2010). Association of apolipoprotein M with high-density lipoprotein kinetics in overweight-obese men. Atherosclerosis.

[10] Dullaart R.P., Plomgaard P., de Vries R., Dahlback B., Nielsen L.B. (2009). Plasma apolipoprotein M is reduced in metabolic syndrome but does not predict intima media thickness. Clinica Chim. Acta.

[11] Plomgaard P., Dullaart R.P., de Vries R., Groen A.K., Dahlback B., Nielsen L.B. (2009). Apolipoprotein M predicts pre-beta-HDL formation: studies in type 2 diabetic and nondiabetic subjects. J. Intern. Med..

[12] Ravnsborg T., Andersen L.L., Trabjerg N.D., Rasmussen L.M., Jensen D.M., Overgaard M. (2016). First-trimester multimarker prediction of gestational diabetes mellitus using targeted mass spectrometry. Diabetologia.

[13] Xu N., Nilsson-Ehle P., Ahren B. (2004). Correlation of apolipoprotein M with leptin and cholesterol in normal and obese subjects. J. Nutr. Biochem..

[14] Wolfrum C., Howell J.J., Ndungo E., Stoffel M. (2008). Foxa2 activity increases plasma high density lipoprotein levels by regulating apolipoprotein M. J. Biol. Chem..

[15] Xu N., Nilsson-Ehle P., Hurtig M., Ahren B. (2004). Both leptin and leptin-receptor are essential for apolipoprotein M expression in vivo. Biochem. Biophys. Res. Commun..

[16] Nojiri T., Kurano M., Tokuhara Y., Ohkubo S., Hara M., Ikeda H. (2014). Modulation of sphingosine-1-phosphate and apolipoprotein M levels in the plasma, liver and kidneys in streptozotocin-induced diabetic mice. J. Diabetes Invest..

[17] Obinata H., Hla T. (2019). Sphingosine 1-phosphate and inflammation. Int. Immunol..

[18] Yao Mattisson I., Christoffersen C. (2021). Apolipoprotein M and its impact on endothelial dysfunction and inflammation in the cardiovascular system. Atherosclerosis.

[19] Li T., Yang L., Zhao S., Zhang S. (2018). Correlation between apolipoprotein M and inflammatory factors in obese patients. Med. Sci. Monit..

[20] Tyden H., Lood C., Jonsen A., Gullstrand B., Kahn R., Linge P. (2019). Low plasma concentrations of apolipoprotein M are associated with disease activity and endothelial dysfunction in systemic lupus erythematosus. Arthritis Res. Ther..

[21] Larsen T.M., Dalskov S., van Baak M., Jebb S., Kafatos A., Pfeiffer A. (2010). The Diet, Obesity and Genes (Diogenes) Dietary Study in eight European countries - a comprehensive design for long-term intervention. Obes. Rev..

[22] Christoffersen C., Ahnstrom J., Axler O., Christensen E.I., Dahlback B., Nielsen L.B. (2008). The signal peptide anchors apolipoprotein M in plasma lipoproteins and prevents rapid clearance of apolipoprotein M from plasma. J. Biol. Chem..

[23] Dehondt H., Marino A., Butruille L., Mogilenko D.A., Nzoussi Loubota A.C., Chavez-Talavera O. (2023). Adipocyte-specific FXR-deficiency protects adipose tissue from oxidative stress and insulin resistance and improves glucose homeostasis. Mol. Metab..

[24] Jimenez V., Munoz S., Casana E., Mallol C., Elias I., Jambrina C. (2013). In vivo adeno-associated viral vector-mediated genetic engineering of white and brown adipose tissue in adult mice. Diabetes.

[25] Tani M., Kawakami A., Nagai M., Shimokado K., Kondo K., Yoshida M. (2007). Sphingosine 1-phosphate (S1P) inhibits monocyte-endothelial cell interaction by regulating of RhoA activity. FEBS Lett..

[26] Blanchard V., Garcon D., Jaunet C., Chemello K., Billon-Crossouard S., Aguesse A. (2020). A high-throughput mass spectrometry-based assay for large-scale profiling of circulating human apolipoproteins. J. Lipid Res..

[27] Klimcakova E., Roussel B., Kovacova Z., Kovacikova M., Siklova-Vitkova M., Combes M. (2011). Macrophage gene expression is related to obesity and the metabolic syndrome in human subcutaneous fat as well as in visceral fat. Diabetologia.

[28] Mootha V.K., Lindgren C.M., Eriksson K.F., Subramanian A., Sihag S., Lehar J. (2003). PGC-1alpha-responsive genes involved in oxidative phosphorylation are coordinately downregulated in human diabetes. Nat. Genet..

[29] Vila I.K., Badin P.M., Marques M.A., Monbrun L., Lefort C., Mir L. (2014). Immune cell Toll-like receptor 4 mediates the development of obesity- and endotoxemia-associated adipose tissue fibrosis. Cell Rep..

[30] Kumaraswamy S.B., Linder A., Akesson P., Dahlback B. (2012). Decreased plasma concentrations of apolipoprotein M in sepsis and systemic inflammatory response syndromes. Crit. Care.

[31] Giles D.A., Ramkhelawon B., Donelan E.M., Stankiewicz T.E., Hutchison S.B., Mukherjee R. (2016). Modulation of ambient temperature promotes inflammation and initiates atherosclerosis in wild type C57BL/6 mice. Mol. Metab..

[32] van der Stelt I., Hoevenaars F., Siroka J., de Ronde L., Friedecky D., Keijer J. (2017). Metabolic response of visceral white adipose tissue of obese mice exposed for 5 Days to human room temperature compared to mouse thermoneutrality. Front. Physiol..

[33] Lac M., Tavernier G., Moro C. (2023). Does housing temperature influence glucose regulation and muscle-fat crosstalk in mice?. Biochimie.

[34] Liu L., Shi Z., Ji X., Zhang W., Luan J., Zahr T. (2022). Adipokines, adiposity, and atherosclerosis. Cell Mol. Life Sci..

[35] Taylor E.B. (2021). The complex role of adipokines in obesity, inflammation, and autoimmunity. Clin. Sci. (Lond)..

[36] Wang M., Luo G.H., Liu H., Zhang Y.P., Wang B., Di D.M. (2019). Apolipoprotein M induces inhibition of inflammatory responses via the S1PR1 and DHCR24 pathways. Mol. Med. Rep..

[37] Zhu B., Luo G.H., Feng Y.H., Yu M.M., Zhang J., Wei J. (2018). Apolipoprotein M protects against lipopolysaccharide-induced acute lung injury via sphingosine-1-phosphate signaling. Inflammation.

[38] Chirinos J.A., Zhao L., Jia Y., Frej C., Adamo L., Mann D. (2020). Reduced apolipoprotein M and adverse outcomes across the spectrum of human heart failure. Circulation.

[39] He Y., Chen J., Ma Y., Chen H. (2022). Apolipoproteins: new players in cancers. Front. Pharmacol..

[40] Kawai T., Autieri M.V., Scalia R. (2021). Adipose tissue inflammation and metabolic dysfunction in obesity. Am. J. Physiol. Cell Physiol..

[41] Chen Y., Wang X., Mai J., Zhao X., Liang Y., Gu M. (2013). C-reactive protein promotes vascular endothelial dysfunction partly via activating adipose tissue inflammation in hyperlipidemic rabbits. Int. J. Cardiol..

[42] Malesza I.J., Malesza M., Walkowiak J., Mussin N., Walkowiak D., Aringazina R. (2021). High-fat, western-style diet, systemic inflammation, and gut microbiota: a narrative review. Cells.

[43] Fritsche K.L. (2015). The science of fatty acids and inflammation. Adv. Nutr..

[44] Kiran S., Rakib A., Kodidela S., Kumar S., Singh U.P. (2022). High-fat diet-induced dysregulation of immune cells correlates with macrophage phenotypes and chronic inflammation in adipose tissue. Cells.

[45] Frances L., Croyal M., Ruidavets J.-B., Maraninchi M., Combes G., Raffin J. (2023). Association of circulating levels of apolipoprotein M and adiponectin with insulin sensitivity in overweight subjects and obese patients with weight loss surgery. medRxiv.

[46] Gao J.J., Hu Y.W., Wang Y.C., Sha Y.H., Ma X., Li S.F. (2015). ApoM suppresses TNF-alpha-Induced expression of ICAM-1 and VCAM-1 through inhibiting the activity of NF-kappaB. DNA Cell Biol..

[47] Hoevenaars F.P., Bekkenkamp-Grovenstein M., Janssen R.J., Heil S.G., Bunschoten A., Hoek-van den Hil E.F. (2014). Thermoneutrality results in prominent diet-induced body weight differences in C57BL/6J mice, not paralleled by diet-induced metabolic differences. Mol. Nutr. Food Res..

[48] Tian X.Y., Ganeshan K., Hong C., Nguyen K.D., Qiu Y., Kim J. (2016). Thermoneutral housing accelerates metabolic inflammation to potentiate atherosclerosis but not insulin resistance. Cell Metab..

[49] Christoffersen C., Federspiel C.K., Borup A., Christensen P.M., Madsen A.N., Heine M. (2018). The apolipoprotein M/S1P Axis controls triglyceride metabolism and Brown fat activity. Cell Rep..

[50] Nga H.T., Moon J.S., Tian J., Lee H.Y., Kim S.H., Lee Y.S. (2021). Interleukin-10 attenuates liver fibrosis exacerbated by thermoneutrality. Front. Med. (Lausanne).

[51] Nunes J.R.C., Smith T.K.T., Ghorbani P., O'Dwyer C., Trzaskalski N.A., Dergham H. (2023). Thermoneutral housing does not accelerate metabolic dysfunction-associated fatty liver disease in male or female C57Bl/6J mice fed a Western diet. Am. J. Physiol. Endocrinol. Metab..

[52] Abboud M., Gordon-Thomson C., Hoy A.J., Balaban S., Rybchyn M.S., Cole L. (2014). Uptake of 25-hydroxyvitamin D by muscle and fat cells. J. Steroid Biochem. Mol. Biol..

[53] Ahnstrom J., Faber K., Axler O., Dahlback B. (2007). Hydrophobic ligand binding properties of the human lipocalin apolipoprotein M. J. Lipid Res..

[54] Elsoe S., Ahnstrom J., Christoffersen C., Hoofnagle A.N., Plomgaard P., Heinecke J.W. (2012). Apolipoprotein M binds oxidized phospholipids and increases the antioxidant effect of HDL. Atherosclerosis.

[55] D'Angelo S., Motti M.L., Meccariello R. (2020). Omega-3 and omega-6 polyunsaturated fatty acids, obesity and cancer. Nutrients.

[56] Mucida D., Cheroutre H. (2007). TGFbeta and retinoic acid intersect in immune-regulation. Cell Adh. Migr..

[57] Yore M.M., Syed I., Moraes-Vieira P.M., Zhang T., Herman M.A., Homan E.A. (2014). Discovery of a class of endogenous mammalian lipids with anti-diabetic and anti-inflammatory effects. Cell.

[58] Kaabia Z., Poirier J., Moughaizel M., Aguesse A., Billon-Crossouard S., Fall F. (2018). Plasma lipidomic analysis reveals strong similarities between lipid fingerprints in human, hamster and mouse compared to other animal species. Sci. Rep..

[59] Croyal M., Billon-Crossouard S., Goulitquer S., Aguesse A., Leon L., Fall F. (2018). Stable isotope kinetic study of ApoM (apolipoprotein M). Arterioscler. Thromb. Vasc. Biol..

[60] Kurano M., Hara M., Tsuneyama K., Sakoda H., Shimizu T., Tsukamoto K. (2014). Induction of insulin secretion by apolipoprotein M, a carrier for sphingosine 1-phosphate. Biochim. Biophys. Acta.

[61] Kurano M., Tsukamoto K., Shimizu T., Kassai H., Nakao K., Aiba A. (2020). Protection against insulin resistance by apolipoprotein M/Sphingosine-1-Phosphate. Diabetes.

[62] Corvera S. (2021). Cellular heterogeneity in adipose tissues. Annu. Rev. Physiol..

[63] Sevvana M., Kassler K., Ahnstrom J., Weiler S., Dahlback B., Sticht H. (2010). Mouse ApoM displays an unprecedented seven-stranded lipocalin fold: folding decoy or alternative native fold?. J. Mol. Biol..

