# Supplementary material for: The adipocyte apolipoprotein M is negatively associated with inflammation
Source: J Lipid Res. 2024 Sep 19;65(10):100648. doi: 10.1016/j.jlr.2024.100648 (PMC11513530; doi:10.1016/j.jlr.2024.100648)
Supplement: Supplemental data [file mmc1.docx]

**Supplemental data**

**Table S1: List of primers and Assays On Demand for gene expression studies.**

| **Gene** | **Primer forward sequence** | **Primer reverse sequence** |
| --- | --- | --- |
| Murine *Tnfα* | CTGAACTTCGGGGTGATCGG | GTGGTTTGCTACGACGTGGG |
| Murine *Il-1β* | CAGGCAGGCAGTATCACTCA | AGGCCACAGGTATTTTGTCG |
| Murine *Il-6* | TCCAGTTGCCTTCTTGGGAC | GTGTAATTAAGCGCCGACTTG |
| Murine *Mcp-1* | ACTGAAGCCAGCTCTCTCTTCCTC | TTCCTTCTTGGGGTCAGCACAGAC |
| Murine *Cd68* | CTTCCCACAGGCAGCACAG | AATGATGAGAGGCAGCAAGAGG |
| Murine *Cd11c* | AGAGCCAGAACTTCCCAACTG | TCTGAAGCTGGCTCATCACAG |
| Murine *Saa3* | GAACTATGATGCTGCCCGGA | TTGGCAAACTGGTCAGCTCT |
| Murine *Hprt* | CAGACTGAAGAGCTACTGTAATGA | CTTTCCAGTTAAAGTTGAGAGATCA |
| Murine *Tbp* | GGAATTGTACCGCAGCTTCAAA | GATGACTGCAGCAAATCGCTT |
| *mcherry* | ATGGTGAGCAAGGGCGAGGA | TCGCCCTCGATCTCGAACTC |
|  | **Assay On Demand number** | |
| Murine *Apom* | Mm00444525_m1 | |
| Human *APOM* | Hs00219533_m1 | |
| Human *TNFα* | Hs00174128_m1 | |
| Human *IL-1β* | Hs00174097_m1 | |
| Human *IL-6* | Hs00985639_m1 | |
| Human *MCP-1* | Hs00234140_m1 | |
| Human *PUM1* | Hs00472881_m1 | |
| Human *LRP10* | Hs01047362_m1 | |
| Human *PSMC4* | Hs00197826_m1 | |

| **Table S2. Anthropometric and clinical characteristics of the 300 individuals.** |
| --- |

|  | Mean (SD) |  |  |
| --- | --- | --- | --- |
| BMI, kg/m^2^ | 34.1 (4.7) |  |  |
| Fat mass, % body weight | 39.3 (7.5) |  |  |
| Waist circumference, cm | 106.0 (12.0) |  |  |
| Triglycerides, g/L | 1.36 (0.64) |  |  |
| Total cholesterol, g/L | 4.93 (0.99) |  |  |
| LDL-C, g/L | 3.05 (0.84) |  |  |
| HDL-C, g/L | 1.25 (0.35) |  |  |
| Fasting glucose, mmol/L | 5.1 (0.6) |  |  |
| Insulin, IU/L | 10.2 (4.9) |  |  |
| HOMA-IR | 2.3 (1.2) |  |  |
| hs-CRP, mg/L | 3.8 (3.3) |  |  |
| Systolic blood pressure, mm Hg | 126 (14) |  |  |
| Diastolic blood pressure, mmHg | 78 (11) |  |  |
| 101 men and 199 women with bioclinical investigation and needle subcutaneous abdominal adipose tissue biopsies.  hs-CRP: high-sensitivity C-reactive protein; HOMA-IR: Homeostasis Model Assessment of Insulin Resistance. | | | |

**Figure S1: Body composition of *Apom^-/-^* mice before and after a 3-month HFD.**

**
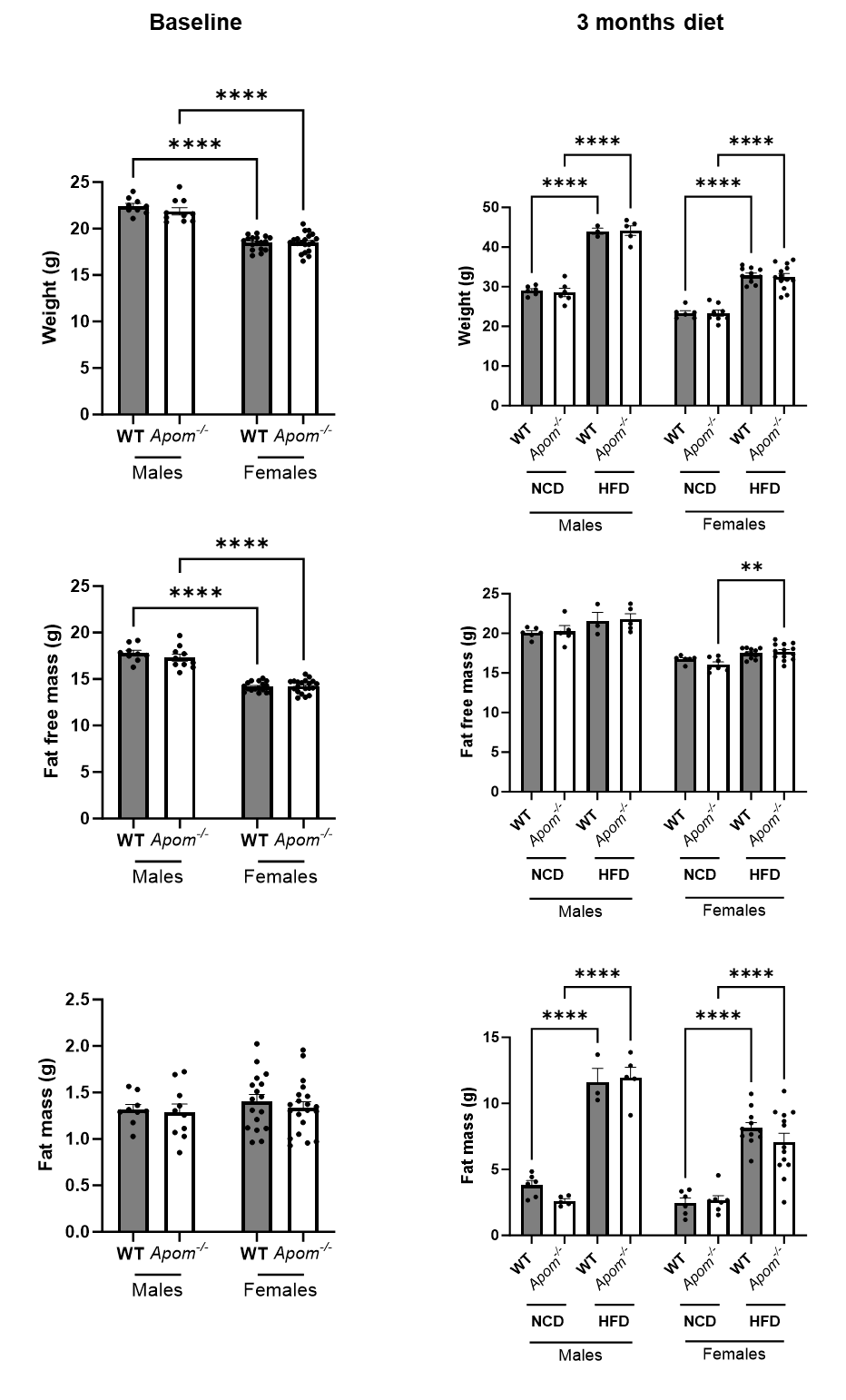
**

Body weight, fat free mass and fat mass of mice before and after 3 months of NCD or HFD (n = 3-13 per group). Grey bars, WT mice; white bars, *Apom^-/-^* mice. Results are presented as mean ± SEM and were analyzed by Mann-Whitney’s test within each group. HFD, high fat diet; NCD, normal chow diet; WT, wildtype.

**Figure S2: Adipocyte size in the perigonadal adipose tissue of *Apom^-/-^* and WT mice after HFD.**

Distribution of the adipocyte sizes in the perigonadal fat pad of mice after 3 months of 60 % HFD. Grey bars represent WT mice (n=21); white bars, *Apom^-/-^* mice (n=24). Results are presented as mean ± SEM and were analyzed by Chi-square test. HFD, high fat diet; WT, wildtype.

**Figure S3: Representative light microscopic image of crown-like structures in the perigonadal adipose tissue of *Apom*^-/-^ and WT mice.**

**
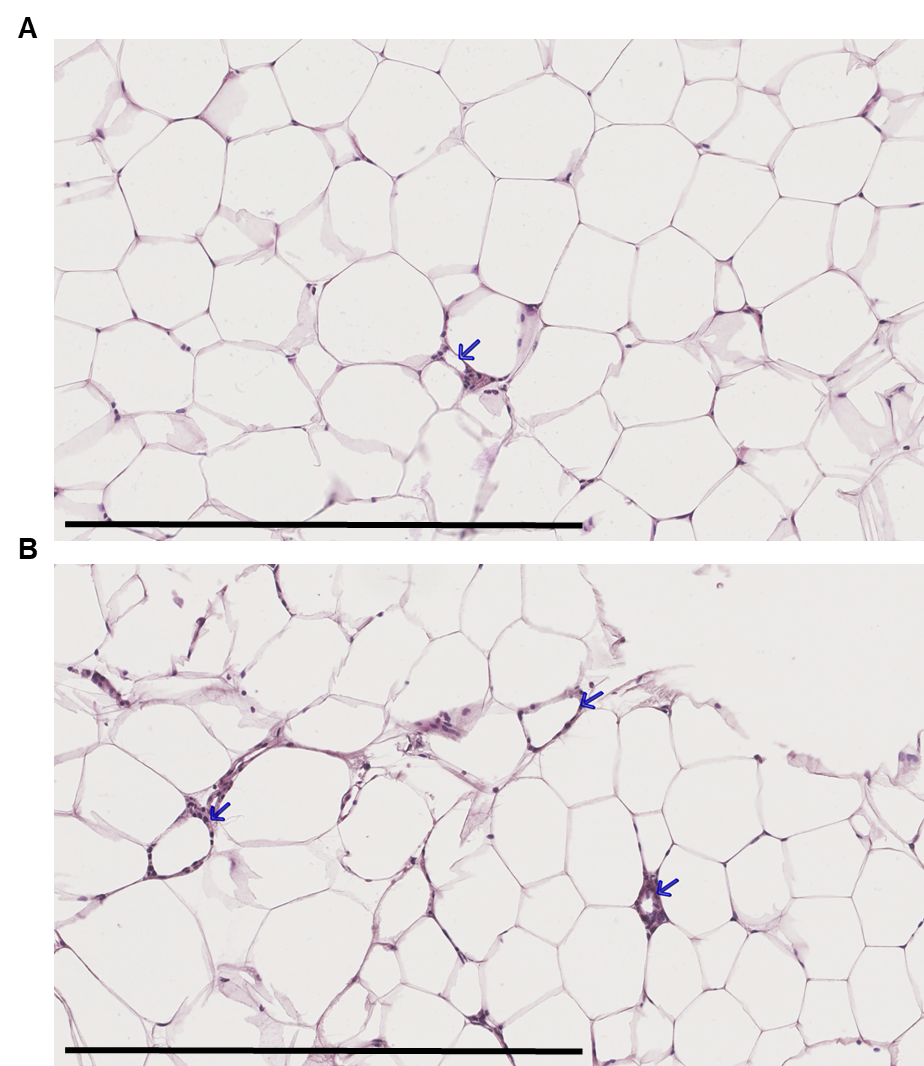
**

Tissue sections were stained with hematoxylin and eosin. A, PGAT of a WT mouse; B, PGAT of an *Apom^-/-^* mouse. Bar: 500 µm. Blue arrows indicate CLS. CLS, crown-like structures; PGAT, perigonadal adipose tissue; WT, wildtype.

**Figure S4: Gating strategy to identify pro-inflammatory macrophages in SVF of perigonadal adipose tissue from *Apom*^-/-^ and WT mice.**


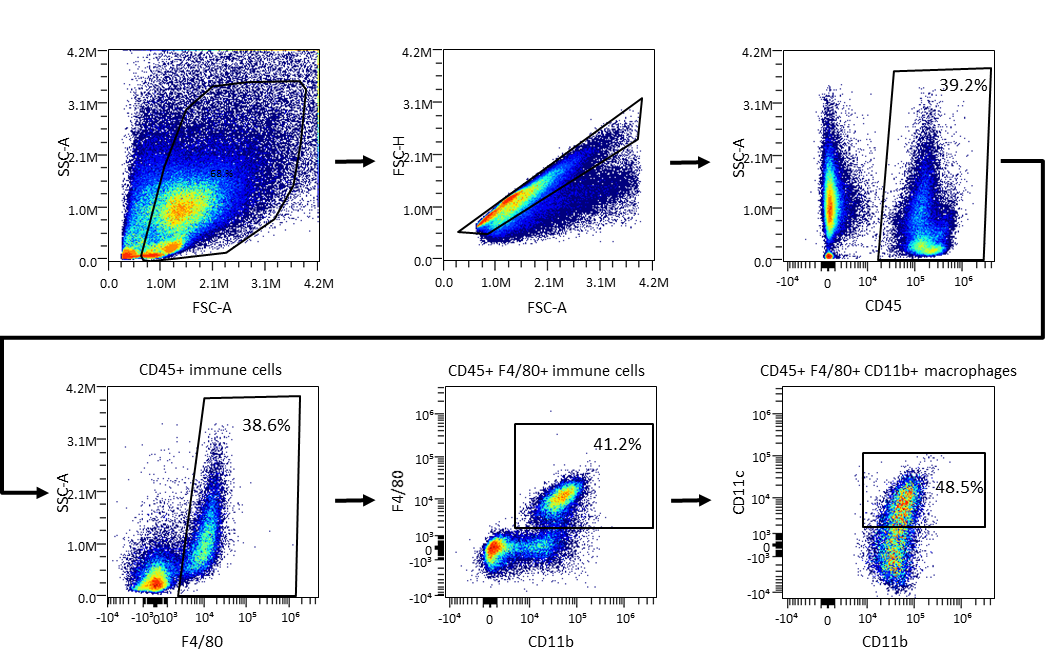


Displayed pictures were acquired for a HFD-fed WT mice. Size and granularity of cells from SVF were analyzed by FSC and SSC using an Aurora Cell Sorter flow cytometer. Pro-inflammatory macrophages were defined as CD45^+^ F4/80^+^ CD11b^+^ CD11c^+^ cells. FSC, forward scatter; HFD, high fat diet; SSC, side scatter; SVF, stromal vascular fraction; WT, wildtype.

**Figure S5: Stain-Free and β-actin images of the Western-blot presented Figure 3B.**


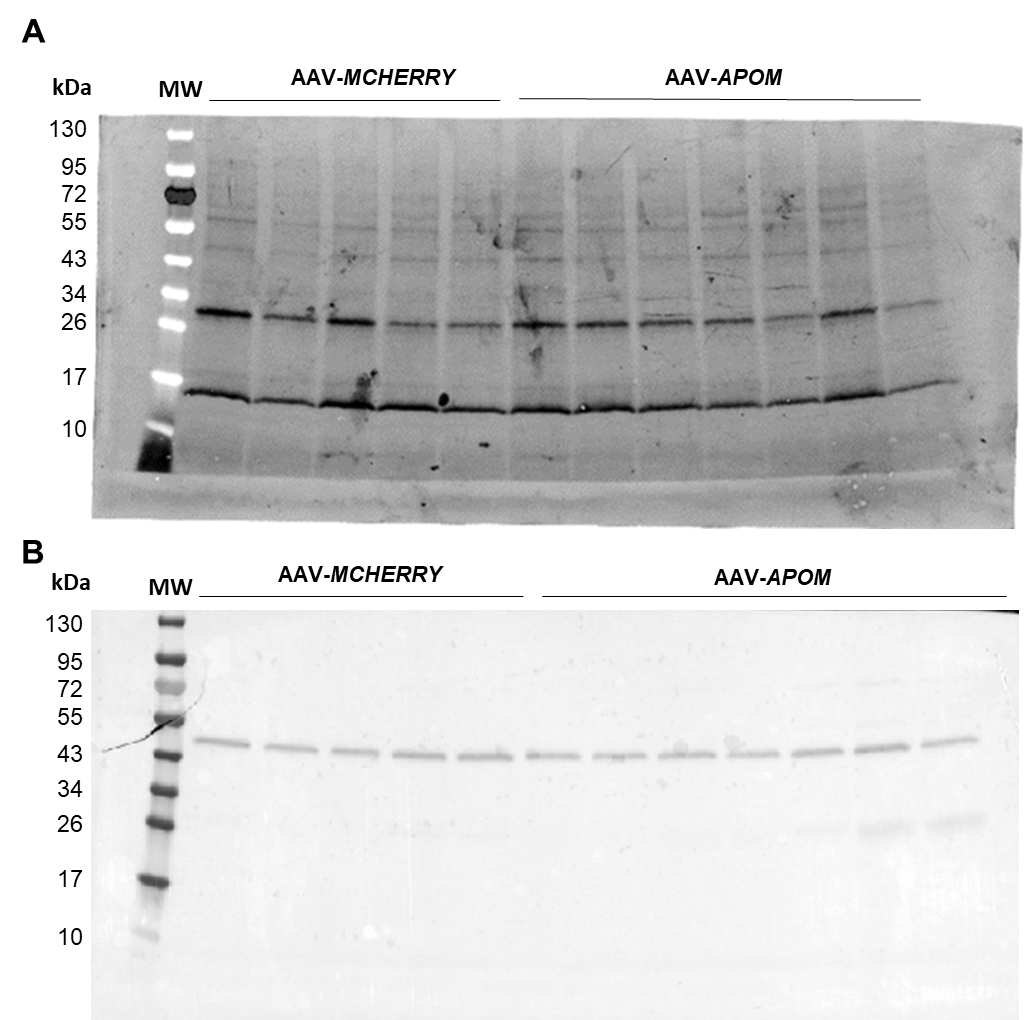


The PGAT of mice were transduced with AAVs encoding MCHERRY (AAV-*MCHERRY,* n = 5) or human APOM (AAV-*APOM*, n = 7), and the mice were fed a HFD for 5 weeks (n = 26-28 per group). 30 µg of PGAT protein were deposited in each lane and separated using a 4-20% SDS-PAGE.

(A) Stain-free blot obtained by UV light exposure using a ChemiDoc imaging system of the blot displayed Figure 3B. (B) The same blot hybridated with anti-β-actin (Cell Signalling Technology, #4970 diluted 1/2000) and anti-APOM (Abnova, #H00055937-M03 diluted 1/1000) antibodies then HRP-conjugated secondary antibodies and vizualized using a Clarity ECL reagent after 3 sec UV light exposure.
